# Chronic Intermittent Hypoxia Disrupts Intestinal Homeostasis Through Gut Microbiota Remodeling and Microbiota-Metabolite Interactions

**DOI:** 10.3390/biom16081186

**Published:** 2026-08-14

**Authors:** Yuying He, Jun Gao, Qiang Li, Chuxi Zhang, Mingrui Zhai, Yuehua Liu

**Affiliations:** 1 Department of Orthodontics, Shanghai Stomatological Hospital & School of Stomatology, Fudan University, Shanghai 200001, China; heyy23@m.fudan.edu.cn (Y.H.); gaojun_kq@fudan.edu.cn (J.G.);; 2 Shanghai Key Laboratory of Craniomaxillofacial Development and Diseases, Fudan University, Shanghai 200001, China

**Keywords:** obstructive sleep apnea, intermittent hypoxia, gut microbiome, intestinal barrier, *Akkermansia muciniphila*

## Abstract

Obstructive sleep apnea (OSA) is characterized by chronic intermittent hypoxia (CIH), which contributes to systemic metabolic disorders. However, the mechanisms underlying CIH-induced intestinal dysfunction remain unclear. In this study, we investigated the effects of CIH on intestinal barrier integrity, gut microbiota, and host metabolism using a multi-omics approach. Male C57BL/6J mice were exposed to six weeks of CIH or normoxia. Colonic barrier integrity was assessed by histological and molecular analyses. Gut microbiota was profiled by full-length 16S rRNA gene sequencing. Untargeted metabolomics was performed on fecal and serum samples, followed by integrated microbiome–metabolome analysis. CIH markedly impaired colonic barrier integrity, as evidenced by disrupted crypt architecture, reduced goblet cell abundance, and decreased expression of ZO-1, Occludin, and Claudin-5. CIH also induced gut microbial dysbiosis, characterized by depletion of the beneficial mucin-associated bacterium *Akkermansia muciniphila* and enrichment of several anaerobic taxa. Metabolomic analysis revealed opposite alterations of PC (20:2/0:0) and LysoPE (20:5/0:0) between feces and serum, whereas melatonin was consistently decreased in both compartments. Integrated multi-omics analysis further revealed close associations between microbial dysbiosis and metabolic remodeling. Collectively, these findings demonstrate that CIH disrupts intestinal homeostasis through coordinated alterations in barrier integrity, gut microbiota composition, and host metabolism, providing new insights into the intestinal mechanisms underlying OSA-associated systemic dysfunction.

## 1. Introduction

Obstructive sleep apnea (OSA) is one of the most common sleep-related breathing disorders and represents an increasing public health burden. Its hallmark pathological feature is chronic intermittent hypoxia (CIH), caused by recurrent upper airway obstruction during sleep [1]. CIH trigger excessive production of reactive oxygen species (ROS), systemic inflammation, and metabolic dysregulation [2]. These pathological processes contribute substantially to the development of cardiovascular diseases, insulin resistance, nonalcoholic fatty liver disease, neurocognitive impairment, and other systemic complications associated with OSA [3,4]. Despite direct hypoxic injury in peripheral organs, recent evidence has demonstrated that intermittent hypoxia-induced gut dysbiosis contributes to cardiovascular dysfunction through microbial metabolite alterations and promotes neuroinflammation via gut-derived immune responses [4,5]. These findings suggest that intestinal dysfunction may represent an important pathogenic contributor to OSA-associated systemic complications. However, the effects of CIH on intestinal homeostasis and the underlying mechanisms remain incompletely understood.

The intestine is not only the largest digestive organ but also a critical mucosal barrier that regulates host immunity and metabolism [6]. Owing to the steep oxygen gradient across the intestinal epithelium, the gut is particularly susceptible to hypoxia–reoxygenation stress [7]. Recurrent hypoxia–reoxygenation impairs intestinal microvascular perfusion and endothelial function, thereby compromising epithelial oxygen supply and barrier integrity [8]. Meanwhile, excessive ROS activate hypoxia- and inflammation-related signaling pathways, such as hypoxia-inducible factor-1α (HIF-1α) and nuclear factor-κB (NF-κB), resulting in epithelial injury and tight junction disruption [9]. Experimental studies have shown that CIH disrupts intestinal barrier integrity by reducing goblet cell numbers, impairing mucus production, decreasing tight junction protein expression, and increasing intestinal permeability [10,11]. Clinical studies have demonstrated increased circulating biomarkers of intestinal permeability, including intestinal fatty acid-binding protein (I-FABP), lipopolysaccharide-binding protein (LBP), and lipopolysaccharide (LPS), in patients with OSA, and these alterations correlate with disease severity and nocturnal hypoxemia [12]. Furthermore, continuous positive airway pressure (CPAP) therapy has been reported to partially improve intestinal barrier function, supporting a causal relationship between intermittent hypoxia and intestinal injury [13]. These findings suggest that intestinal barrier dysfunction may be an important pathological consequence of CIH.

The gut microbiota plays a pivotal role in maintaining intestinal homeostasis. Because microbial communities are highly sensitive to oxygen availability, repeated hypoxia–reoxygenation cycles during CIH can reshape gut microbial composition [14]. Experimental studies have reported depletion of beneficial bacteria, such as *Akkermansia*, together with enrichment of anaerobic and opportunistic taxa following CIH exposure [15,16,17]. Among these, *Akkermansia muciniphila* (*A. muciniphila*) has attracted considerable attention as a next-generation probiotic owing to its essential role in preserving mucus layer integrity, strengthening epithelial barrier function, regulating host immune responses, and improving metabolic homeostasis [18]. Recent studies further emphasize that depletion of *A. muciniphila* is closely associated with obesity, diabetes, inflammatory bowel disease, and several chronic inflammatory disorders [19].

Importantly, the biological effects of gut dysbiosis are largely mediated through microbial metabolites. Short-chain fatty acids, bile acids, phospholipids, indole derivatives, and other microbial metabolites have been reported to regulate epithelial integrity, immune responses, and host metabolism [20,21,22]. Serum metabolomics studies in patients with OSA have identified widespread alterations in lipid, amino acid, bile acid, and energy metabolism, indicating that intermittent hypoxia is accompanied by systemic metabolic remodeling [23]. Under conditions of impaired intestinal barrier function, these metabolites may enter the circulation and contribute to systemic metabolic remodeling [24]. However, the integrated relationships among gut microbial dysbiosis, metabolic alterations, and intestinal barrier dysfunction during CIH remain poorly understood.

We therefore hypothesized that CIH disrupts intestinal homeostasis by remodeling the gut microbiota and its associated metabolic network, thereby impairing intestinal barrier integrity and contributing to systemic metabolic alterations. To test this hypothesis, we established a six-week mouse model of CIH and systematically evaluated CIH-induced intestinal alterations by integrating histopathological analysis, full-length 16S rRNA sequencing, untargeted fecal and serum metabolomics, and assessment of tight junction proteins. Furthermore, cross-compartment metabolite profiling and microbiota-metabolite correlation analyses were performed to identify functional interactions between gut microorganisms and host metabolism. Our findings provide new insights into the gut microbiota-metabolite axis underlying CIH-induced intestinal barrier dysfunction and may facilitate the development of microbiota-targeted therapeutic strategies for OSA-related complications.

## 2. Methods

### 2.1. Animals and CIH Exposure

Specific-pathogen-free (SPF) male C57BL/6J mice (6–8 weeks old, weighed 22.6 ± 1.1 g at baseline) were supplied by Shanghai SLAC Laboratory Animal Co., Ltd. (Shanghai, China). Sample size was determined based on previous studies using similar CIH mouse models [25]. Animals were maintained in a standardized barrier facility under controlled environmental conditions, including a temperature of 22 ± 1 °C, relative humidity of 50% ± 5%, and a 12 h light/dark cycle. All animal experiments were performed following the guidelines established by the Laboratory Animal Center of Shanghai Jiao Tong University and were reviewed and approved by the Institutional Animal Care and Use Committee (Approval No. A2024502).

After acclimation, mice were randomly allocated into normoxia (NOR) and CIH groups using a computer-generated random number sequence. Five mice were housed per individually ventilated cage. Bedding, chow, water, and cage changing schedules were identical between groups to minimize cage effects on gut microbiota composition. CIH exposure was performed using an automated oxygen regulation system (OxyCycler; BioSpherix, Parish, NY, USA) according to previously reported procedures [25,26]. Briefly, animals were placed in sealed exposure chambers and subjected to repeated oxygen oscillations during the light phase (08:00–17:00). The oxygen profile consisted of intermittent hypoxic episodes (5–6 ± 1% O_2_) lasting approximately 30 s, followed by recovery to room air oxygen levels (21% O_2_). This cycle was repeated for 8 h per day over a 6-week period. Mice in the NOR group remained in the same chamber environment with stable oxygen concentration and mechanical noise, and received identical handling procedures to control for nonspecific effects associated with chamber exposure. No animals died or were excluded during the study. This study is reported in accordance with the ARRIVE guidelines.

### 2.2. Pulse Oximetry

Following the completion of the 6-week CIH protocol, arterial oxygen saturation (SaO_2_) and heart rate (HR) were monitored in awake mice during a resting state. To ensure optimal signal transmission, fur around the neck of each animal was depilated prior to measurement. Physiological parameters were continuously recorded for 15 min using the MouseOx Plus pulse oximeter system (STARR Life Sciences, Oakmont, PA, USA) equipped with a non-invasive throat collar sensor, following a 15-min acclimation period. Only successful data points devoid of error codes were utilized for subsequent analysis. For each subject, the mean SaO_2_, minimum SaO_2_, and average HR were calculated over the entire 15-min evaluation window.

### 2.3. Tissue Collection and Sample Preparation

At the end of the experimental period, mice were anesthetized and euthanized following approved animal protocols. Blood samples were collected by cardiac puncture, and serum was obtained by centrifugation and stored at −80 °C until further analysis. Fresh fecal pellets were collected individually before sacrifice, immediately frozen, and stored at −80 °C for microbiome and metabolomic analyses. Approximately 1 cm of distal colon tissue was carefully dissected after euthanasia and rinsed with ice-cold PBS to remove residual contents. Tissue samples were divided for subsequent histological and molecular analyses. Samples for biochemical assays were snap-frozen in liquid nitrogen and stored at −80 °C, while tissues for histological evaluation were fixed in 4% paraformaldehyde.

### 2.4. Full-Length 16S rRNA Gene Sequencing

Fecal samples were collected and stored at −80 °C until analysis. Total microbial DNA was extracted using the FastPure Stool DNA Isolation Kit (MJYH, Shanghai, China). DNA concentration and purity were determined using a NanoDrop 2000 spectrophotometer (Thermo Scientific, Waltham, MA, USA), and DNA integrity was assessed by 1% agarose gel electrophoresis. The full-length 16S rRNA gene was amplified using universal primers 27F (5′-AGRGTTYGATYMTGGCTCAG-3′) and 1492R (5′-RGYTACCTTGTTACGACTT-3′) with PacBio barcode sequences. Purified PCR products were pooled for SMRTbell library construction and sequenced on the PacBio Sequel IIe platform by Majorbio Bio-Pharm Technology Co., Ltd. (Shanghai, China).

Raw sequences were demultiplexed according to the barcode sequences assigned to individual samples, followed by orientation correction and length filtering. For full-length 16S rRNA gene analysis, sequences ranging from 1000 to 1800 bp were retained. The quality-controlled sequences were subsequently denoised using the DADA2_CCS plugin implemented in the QIIME2 pipeline with default parameters to generate amplicon sequence variants (ASVs). All samples were rarefied to 8530 sequences. Following rarefaction, a total of 2171 ASVs were obtained across the analyzed samples. Good’s coverage exceeded 0.99 for all samples, indicating adequate sequencing coverage. Taxonomic classification was performed using the classify–consensus–blast method implemented in QIIME2 against the NT_Taxon_core_v2024/16s_bacteria reference database. Alpha diversity was evaluated using Shannon diversity indices. Beta diversity was assessed based on Bray–Curtis distances and visualized by principal coordinate analysis (PCoA). Microbial composition was compared based on relative abundance and visualized using heatmap analysis. Differentially abundant taxa between groups were identified using linear discriminant analysis effect size (LEfSe) analysis with an LDA score > 3 and *p* < 0.05 as significance criteria.

### 2.5. Untargeted Fecal and Serum Metabolomic Profiling

Fecal and serum samples were stored at −80 °C until metabolomic analysis. For metabolite extraction, 100 mg fecal samples were homogenized with 800 μL methanol/water (4:1, *v*/*v*) extraction solution containing four internal standards, including L-2-chlorophenylalanine (0.02 mg/mL). Serum samples (100 μL) were extracted with 400 μL acetonitrile/methanol (1:1, *v*/*v*) containing internal standards. After low-temperature ultrasonic extraction and centrifugation (13,000× *g*, 15 min, 4 °C), the supernatants were collected for LC-MS/MS analysis. Equal volumes of extracts from all samples were pooled to prepare quality control (QC) samples. Three QC samples were included in the analytical sequence, and one QC sample was injected after every 5 study samples to monitor analytical stability, reproducibility, and instrumental performance throughout the analytical sequence.

Metabolomic profiling was performed using a UHPLC system coupled with an Orbitrap Exploris 240 mass spectrometer equipped with an ESI source (Majorbio Bio-Pharm Technology Co., Ltd., Shanghai, China). Chromatographic separation was achieved using an ACQUITY HSS T3 column (100 × 2.1 mm, 1.8 μm; Waters, Milford, MA, USA). Data were acquired in both positive and negative ion modes. After data acquisition, raw data were processed using Progenesis QI (version 4.0; Waters Corporation, Milford, MA, USA) for peak detection, extraction, alignment, and integration. Metabolites were annotated by matching MS/MS spectra against HMDB, METLIN, and Majorbio in-house databases. The feature matrix was subjected to quality-control preprocessing. Features with >20% missing values in any experimental group were removed; the remaining missing entries were imputed with the global minimum across all samples. After total-sum normalization, variables with a relative standard deviation (RSD) > 30% in QC samples were excluded. The final matrix was log10-transformed prior to statistical analysis. After data normalization and quality control filtering, principal component analysis (PCA) and partial least squares discriminant analysis (PLS-DA) were performed. Differential metabolites were identified based on variable importance in projection (VIP) > 1 and nominal *p* < 0.05. FDR-adjusted *q* values were additionally evaluated. Differential metabolites were visualized using volcano plots and hierarchical clustering heatmaps. KEGG pathway enrichment analysis was performed to identify metabolic pathways associated with CIH-induced alterations.

### 2.6. Integrated Analysis of Gut Microbiota and Metabolomics

Differential metabolites identified from fecal and serum metabolomic datasets were first compared to determine overlapping metabolic features associated with CIH treatment. Spearman correlation analysis was performed between significantly altered microbial taxa and differential metabolites to evaluate their potential associations. Correlation networks were constructed based on statistically significant microbial–metabolite relationships. Associations were considered significant when the absolute correlation coefficient was |r| > 0.6 and *p* < 0.05. The identified microbial–metabolite interactions were visualized using correlation heatmaps and Circos plots to illustrate the relationships between key bacterial taxa and metabolic signatures.

### 2.7. Histological Analysis of Colonic Tissues

Colonic tissues were fixed in 4% paraformaldehyde for 24 h, dehydrated through a graded ethanol series, embedded in paraffin, and sectioned into 4-μm-thick slices. For hematoxylin and eosin (HE) staining, tissue sections were deparaffinized, rehydrated, and stained according to standard histological procedures. Histopathological changes were evaluated under a light microscope, with particular attention to crypt architecture, epithelial integrity, and inflammatory cell infiltration.

For Alcian blue staining, paraffin sections were deparaffinized, rehydrated, and stained with Alcian blue solution (C0155, pH2.5, Beyotime, Shanghai, China) to visualize acidic mucins in goblet cells, followed by routine nuclear counterstaining. Images were captured under identical microscope settings. Goblet cell abundance was quantified by measuring the Alcian blue-positive area using ImageJ software (version 1.54) and expressed as the percentage of positively stained area relative to the crypt area. At least five randomly selected fields were analyzed for each section, and the average value was used for statistical analysis. ImageJ quantification was performed independently by two investigators blinded to the experimental groups.

### 2.8. Western Blot Analysis

Total protein was extracted from colonic tissues using ice-cold RIPA lysis buffer supplemented with protease and phosphatase inhibitors. After centrifugation at 12,000× *g* for 15 min at 4 °C, protein concentrations were determined using a BCA Protein Assay Kit (KTD3001; Abbkine, Wuhan, China). Equal amounts of protein were separated by SDS-PAGE on 4–20% precast gradient gels and transferred onto PVDF membranes. The membranes were blocked with 5% non-fat milk in TBST for 1 h at room temperature and incubated overnight at 4 °C with primary antibodies against ZO-1 (rabbit, 1:5000, 21773-1-AP; Proteintech, Wuhan, China), Occludin (rabbit, 1:5000, 27260-1-AP; Proteintech), Claudin-5 (rabbit, 1:5000, 29767-1-AP; Proteintech), and β-actin (mouse, 1:20,000, 66009-1-Ig; Proteintech). After washing with TBST, membranes were incubated with HRP-conjugated secondary antibodies (1:20,000) for 2 h at room temperature. Protein bands were detected using an enhanced chemiluminescence (ECL) reagent, quantified with ImageJ software, and normalized to β-actin.

### 2.9. Immunofluorescence Staining

Paraffin-embedded colonic tissue sections (4 μm thick) were deparaffinized, rehydrated, and subjected to heat-induced antigen retrieval. After blocking with 5% bovine serum albumin (BSA) for 1 h, sections were incubated overnight at 4 °C with rabbit primary antibodies against either Occludin (1:200; Proteintech) or ZO-1 (1:200; Proteintech). Following PBS washes, the sections were incubated with an Alexa Fluor 594-conjugated goat anti-rabbit IgG secondary antibody (1:500; Proteintech) for 2 h at room temperature in the dark. Nuclei were counterstained with DAPI, and images were acquired using an Olympus VS200 slide scanner (Olympus, Tokyo, Japan) under identical acquisition parameters. The mean fluorescence intensity of randomly selected fields from each section was quantified using ImageJ, and the average values were utilized for subsequent statistical analysis. ImageJ quantification was performed independently by two investigators blinded to the experimental groups.

### 2.10. Statistical Analysis

Data are presented as mean ± standard deviation (SD). Statistical analyses were performed using GraphPad Prism (version 9.0). The assumptions of normality and homogeneity of variance were verified before applying parametric tests using the Shapiro–Wilk test and Levene’s test, respectively. For comparisons between two groups, an unpaired two-tailed Student’s *t*-test was applied. Detailed statistical procedures regarding microbiome and metabolomic data processing are provided in their respective subsections. Correlation analyses were performed using Spearman’s correlation test. Statistical significance was defined as a two-sided *p* value < 0.05. Significance levels are denoted as: ns, not significant; * *p* < 0.05; ** *p* < 0.01; *** *p* < 0.001.

## 3. Results

### 3.1. CIH Induces Colonic Structural Disruption and Mucus Barrier Impairment

**Figure 1:**
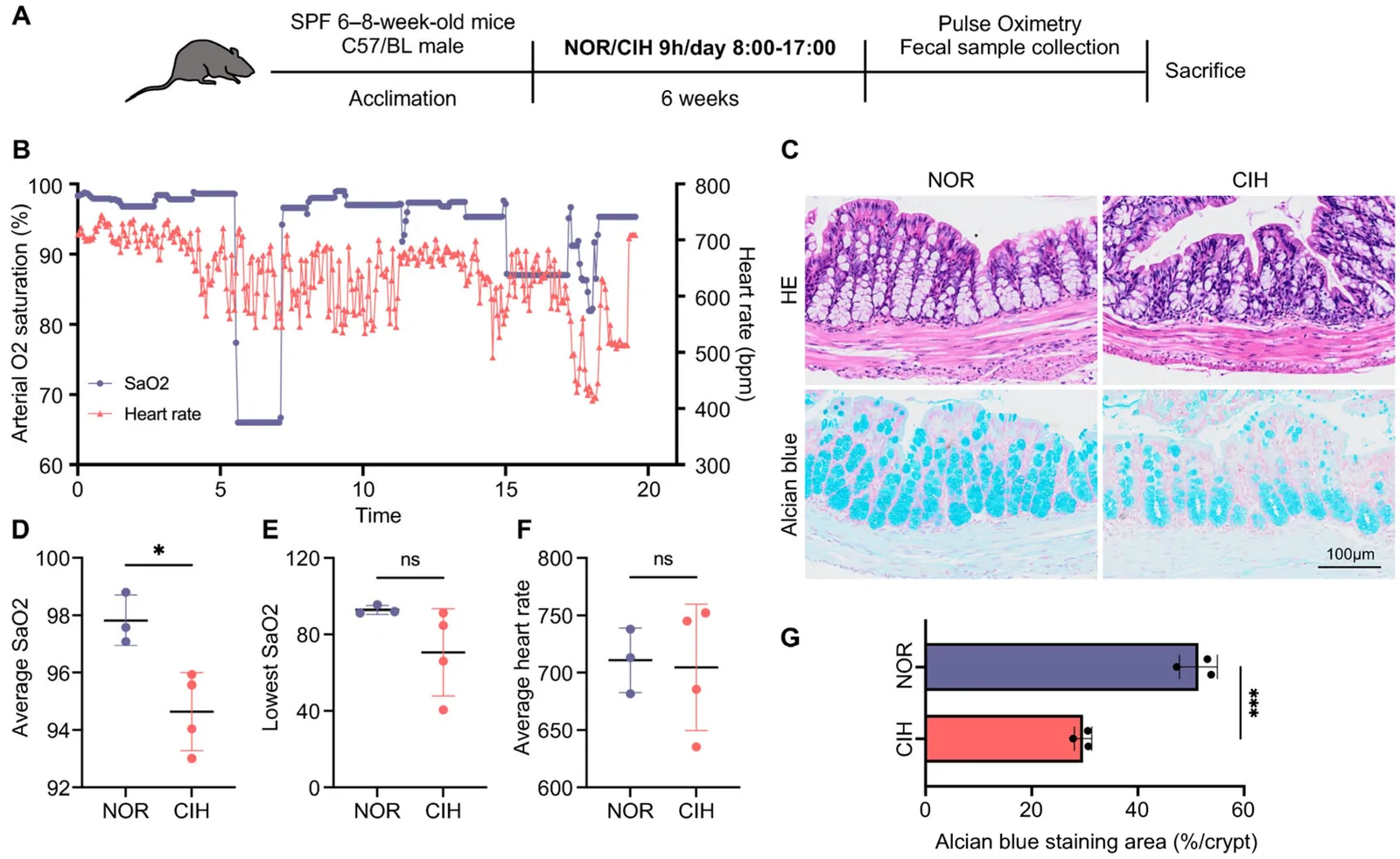
Chronic intermittent hypoxia (CIH) induces systemic hypoxemia and impairs colonic histological integrity. (**A**) Timeline of the experimental design. (**B**) Representative recordings of arterial oxygen saturation (SaO_2_) and heart rate in normoxia (NOR) and CIH mice after 6 weeks of exposure. (**C**) Representative HE staining and Alcian blue staining of colonic tissues from NOR and CIH mice. (**D**) Quantification of mean SaO_2_ (*n* = 3–4). (**E**) Quantification of the lowest arterial oxygen saturation recorded during the monitoring period (*n* = 3–4). (**F**) Quantification of mean heart rate (*n* = 3–4). (**G**) Quantification of Alcian blue-positive area (goblet cells) within colonic crypts (*n* = 3). Data are presented as mean ± SD. Statistical significance was determined using an unpaired two-tailed Student’s *t*-test. * *p* < 0.05, *** *p* < 0.001; ns, not significant.

Mice were first subjected to a 6-week CIH protocol, after which arterial oxygen saturation was monitored to evaluate the effectiveness of the model. During the observation period, mice in the CIH group exhibited a marked reduction in arterial oxygen saturation compared with NOR controls (Figure 1A). The mean SaO_2_ was significantly lower in CIH mice than in NOR mice (*p* = 0.0174) (Figure 1C). Notably, the lowest oxygen saturation recorded in CIH mice reached 40.5% (Figure 1D), whereas the minimum SaO_2_ in NOR mice remained above 90% throughout the monitoring period. In contrast, no significant difference in mean heart rate was observed between the CIH and NOR groups (Figure 1E).

We next investigated the effects of CIH on colonic histopathology. Following the 6-week exposure, mice were euthanized, and colonic tissues were harvested for HE and Alcian blue staining. HE staining revealed disrupted crypt architecture in the colons of CIH mice, characterized by irregular crypt arrangement, partial crypt loss, and mild inflammatory cell infiltration (Figure 1B). Alcian blue staining was subsequently performed to evaluate intestinal mucus production by quantifying the Alcian blue-positive area within colonic crypts. Quantitative analysis demonstrated a significant reduction in Alcian blue-positive goblet cells within the colonic crypts of CIH mice (*p* = 0.0007), indicating impaired intestinal mucus barrier integrity following CIH exposure (Figure 1F).

### 3.2. CIH Reshapes the Gut Microbial Community in Mice

**Figure 2:**
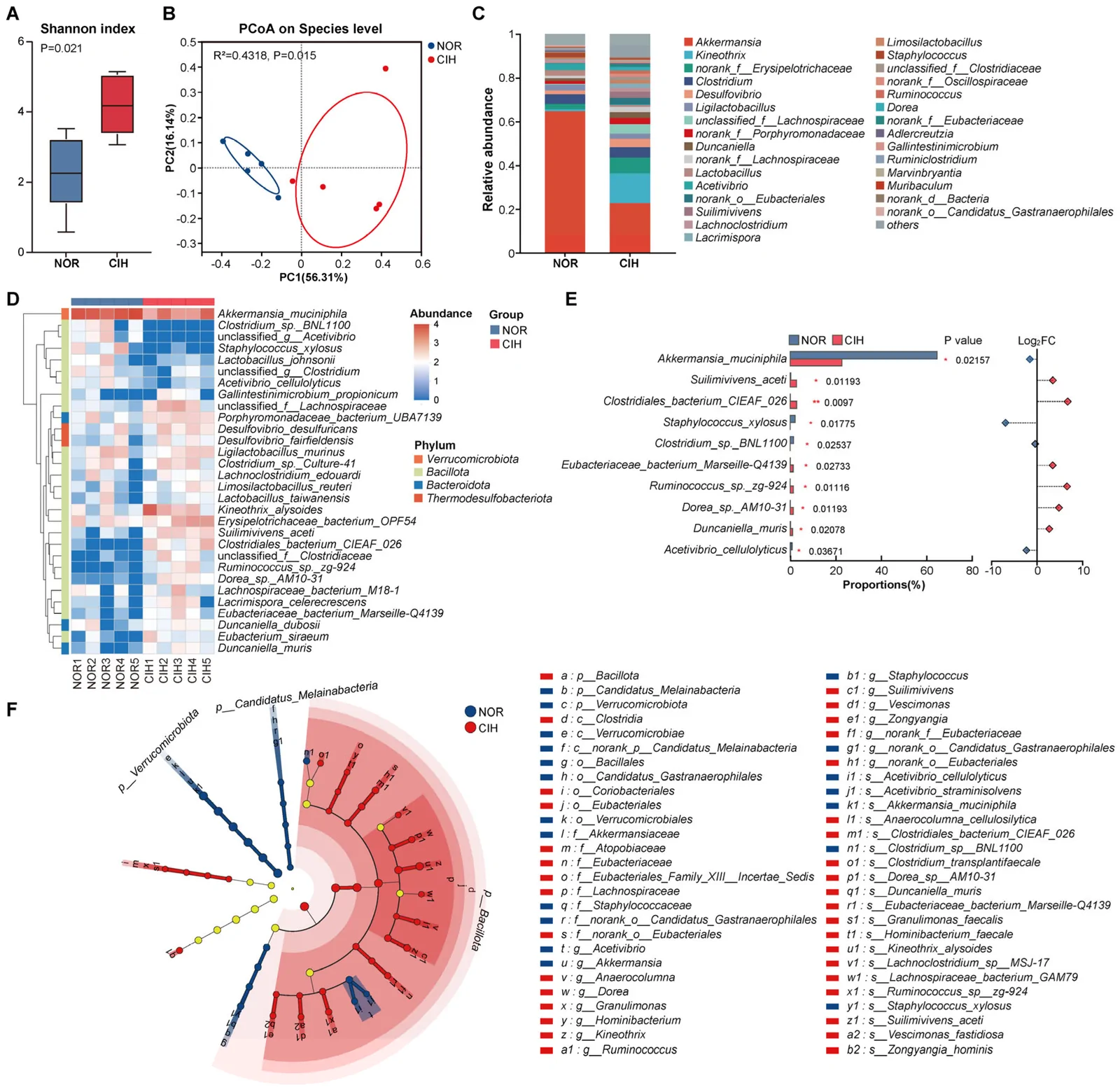
Gut microbiota remodeling following CIH exposure. (**A**) Alpha diversity analysis of gut microbiota based on full-length 16S rRNA sequencing, evaluated by Shannon indices. (**B**) Beta diversity analysis showing microbial community separation between NOR and CIH groups. (**C**) Relative abundance of representative differential bacterial genera between NOR and CIH mice. (**D**) Heatmap showing the relative abundance of the top 30 bacterial genera across samples. (**E**) Comparison of the 10 most abundant bacterial species between NOR and CIH groups via Student’s *t*-test. (**F**) LEfSe analysis identifying differentially enriched bacterial taxa between NOR and CIH groups (LDA score > 3). *n* = 5 per group. Data are presented as mean ± SD. * *p* < 0.05, ** *p* < 0.01.

Subsequently, full-length 16S rRNA sequencing was performed to investigate alterations in the gut microbiota of NOR and CIH mice. Alpha and beta diversity analyses revealed significant shifts in the gut microbial community following CIH exposure (Figure 2A,B). For instance, the genus *Akkermansia*, which was predominant in the gut of NOR mice, was significantly depleted in CIH mice (FC = 0.35, *p* = 0.02157). Conversely, obligate anaerobic genera that were less abundant in the NOR group, such as *Kineothrix* (FC = 13.63, *p* = 0.0601) and *Suilimivivens* (FC = 11.65, *p* = 0.01193), were enriched in the CIH group (Figure 2C). Consistently, a heatmap displaying the relative abundances of the top 30 bacterial genera confirmed that CIH exerted substantial alterations in gut microbial community composition (Figure 2D).

Student’s *t*-test was further performed on the top 10 most abundant species (Figure 2E). Compared with NOR mice, *A. muciniphila* was markedly depleted in the CIH group (FC = 0.35, *p* = 0.0216), accompanied by reduced abundances of *Staphylococcus xylosus*, *Clostridium* sp. *BNL1100*, and *Acetivibrio cellulolyticus*. Conversely, several bacterial species, including *Suilimivivens aceti*, *Clostridiales bacterium CIEAF 026*, *Eubacteriaceae bacterium Marseille-Q4139*, *Ruminococcus* sp. *zg-924*, *Dorea* sp. *AM10-31*, and *Duncaniella muris*, were significantly enriched in CIH mice.

LEfSe analysis was performed to identify bacterial taxa that were differentially enriched between the NOR and CIH groups (LDA score > 3) (Figure 2F). Compared with NOR mice, the CIH group exhibited significant enrichment of the phylum *Bacillota*, together with its downstream taxa including *Clostridia*, *Eubacteriales*, *Lachnospiraceae*, *Eubacteriaceae*, and genera such as *Dorea*, *Granulimonas*, *Hominibacterium*, *Kineothrix*, and *Ruminococcus*. Several species, including *Dorea* sp., *Ruminococcus* sp., *Granulimonas faecalis*, and *Kineothrix alysoides*, were also identified as characteristic biomarkers of the CIH microbiota. In contrast, the NOR group was characterized by enrichment of the phylum *Verrucomicrobiota*, particularly the family *Akkermansiaceae*, genus *Akkermansia*, and the beneficial species *A. muciniphila.* In addition, taxa belonging to *Candidatus Melainabacteria*, *Verrucomicrobiales*, *Acetivibrio*, and *Staphylococcus* were preferentially enriched in NOR mice. These findings indicate that CIH markedly reshaped the gut microbial composition and promoted the expansion of taxa associated with anaerobic fermentation while reducing beneficial mucin-degrading bacteria.

### 3.3. CIH Exposure Induces Alterations in Fecal and Serum Metabolic Profiles

**Figure 3:**
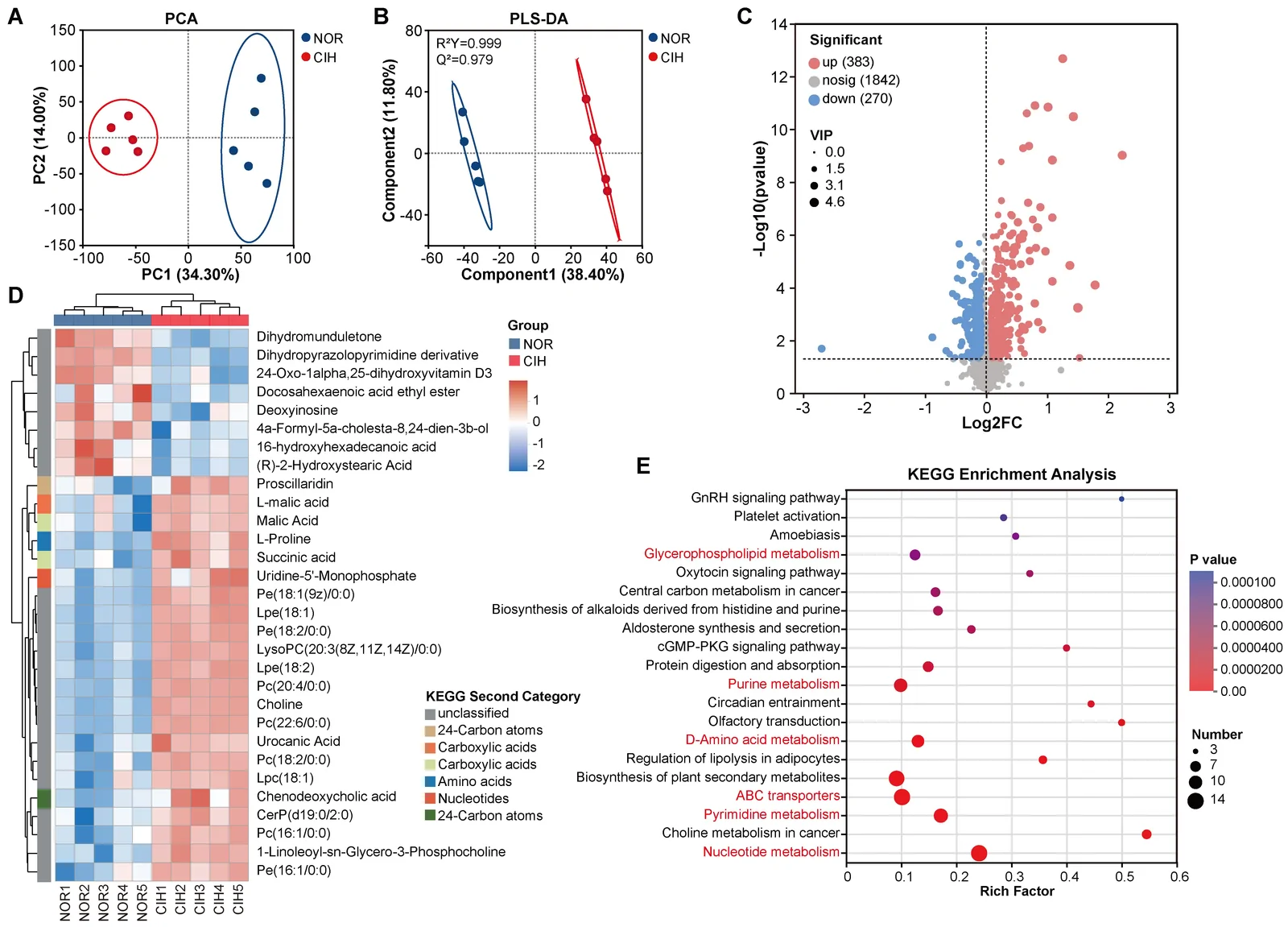
Fecal metabolomic profiling reveals metabolic alterations induced by CIH. (**A**) PCA of fecal metabolomic profiles from NOR and CIH mice. (**B**) PLS-DA showing separation between the two groups. (**C**) Volcano plot showing differentially abundant metabolites between NOR and CIH groups. (**D**) Hierarchical clustering heatmap of the top 30 differential fecal metabolites. (**E**) Kyoto Encyclopedia of Genes and Genomes (KEGG) pathway enrichment analysis of differential fecal metabolites. *n* = 5 per group.

**Figure 4:**
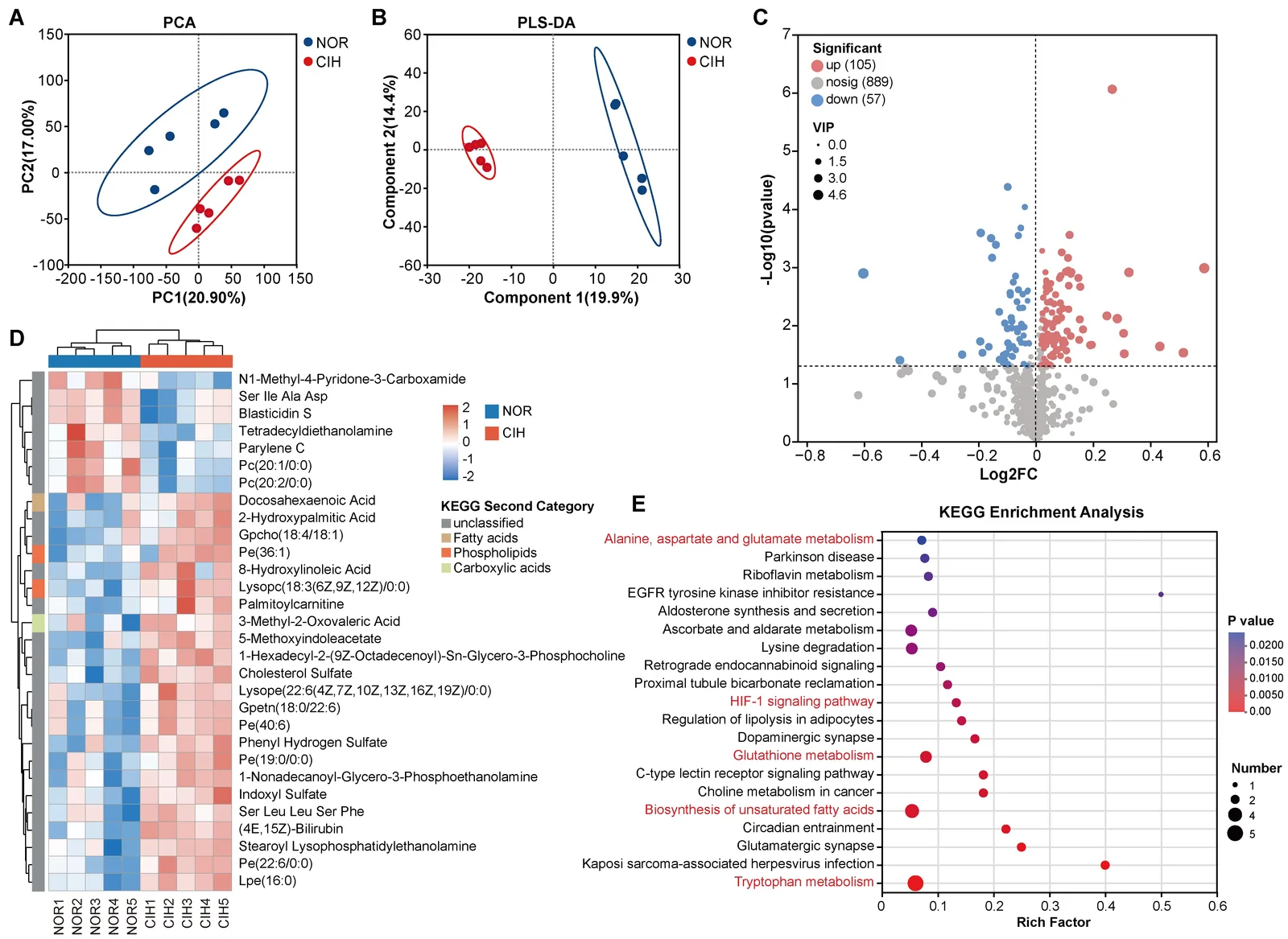
Serum metabolomic profiling reveals systemic metabolic remodeling following CIH. (**A**) PCA of serum metabolomic profiles from NOR and CIH mice. (**B**) PLS-DA showing metabolic separation between NOR and CIH groups. (**C**) Volcano plot displaying differentially abundant serum metabolites. (**D**) Hierarchical clustering heatmap of the top 30 differential serum metabolites. (**E**) KEGG pathway enrichment analysis of differential serum metabolites. *n* = 5 per group.

To investigate the metabolic consequences of gut microbiota remodeling induced by CIH exposure, untargeted metabolomic profiling was performed in fecal and serum samples. PCA and PLS-DA analyses demonstrated clear separation between NOR and CIH groups, indicating substantial alterations in metabolic profiles (Figure 3A,B and Figure 4A,B). Differential metabolite analysis revealed 383 upregulated and 270 downregulated metabolites in fecal samples (Figure 3C), while 105 metabolites were increased and 57 were decreased in serum samples (Figure 4C). Hierarchical clustering based on the top 30 differential metabolites further confirmed distinct metabolic signatures between the two groups, with good intra-group reproducibility (Figure 3D and Figure 4D).

CIH exposure resulted in marked alterations in microbial-associated metabolites in fecal samples (Figure 3D). Specifically, CIH mice showed increased abundance of organic acids (e.g., L-malic acid and succinic acid), amino acid and nucleotide metabolites (e.g., L-proline and uridine-5′-monophosphate), and glycerophospholipid-related metabolites, including phosphatidylcholines (PCs), phosphatidylethanolamines (PEs), and lysophospholipid derivatives. KEGG enrichment analysis indicated that fecal metabolic alterations were particularly associated with glycerophospholipid metabolism, consistent with the pronounced alterations in PCs, PEs, and lysophospholipid derivatives observed in fecal samples, indicating that CIH may perturb intestinal membrane lipid homeostasis (Figure 3E).

Serum metabolomic profiling further revealed systemic metabolic disturbances following CIH exposure (Figure 4C). CIH mice exhibited significant alterations in lipid metabolism, characterized by increased levels of fatty acid-related metabolites, including docosahexaenoic acid, 2-hydroxypalmitic acid, and 8-hydroxylinoleic acid. In addition, CIH exposure resulted in the accumulation of membrane lipid-associated metabolites, such as PCs, PEs, lysophospholipids, and palmitoylcarnitine. KEGG pathway analysis highlighted alterations in HIF-1 signaling, glutathione metabolism, and tryptophan metabolism (Figure 4E). The enrichment of HIF-1 signaling is consistent with the hypoxic conditions imposed by CIH, while alterations in glutathione and tryptophan metabolism may reflect changes in systemic redox homeostasis and metabolic pathways related to neuroactive and circadian regulation, respectively. Collectively, these findings indicate that CIH induces metabolic remodeling in both the intestinal and systemic compartments, characterized by alterations in lipid homeostasis, hypoxia-related signaling, and metabolic stress responses.

### 3.4. Cross-Compartment Metabolite Profiling Identifies Gut Microbiota-Associated Metabolic Remodeling Following CIH

**Figure 5:**
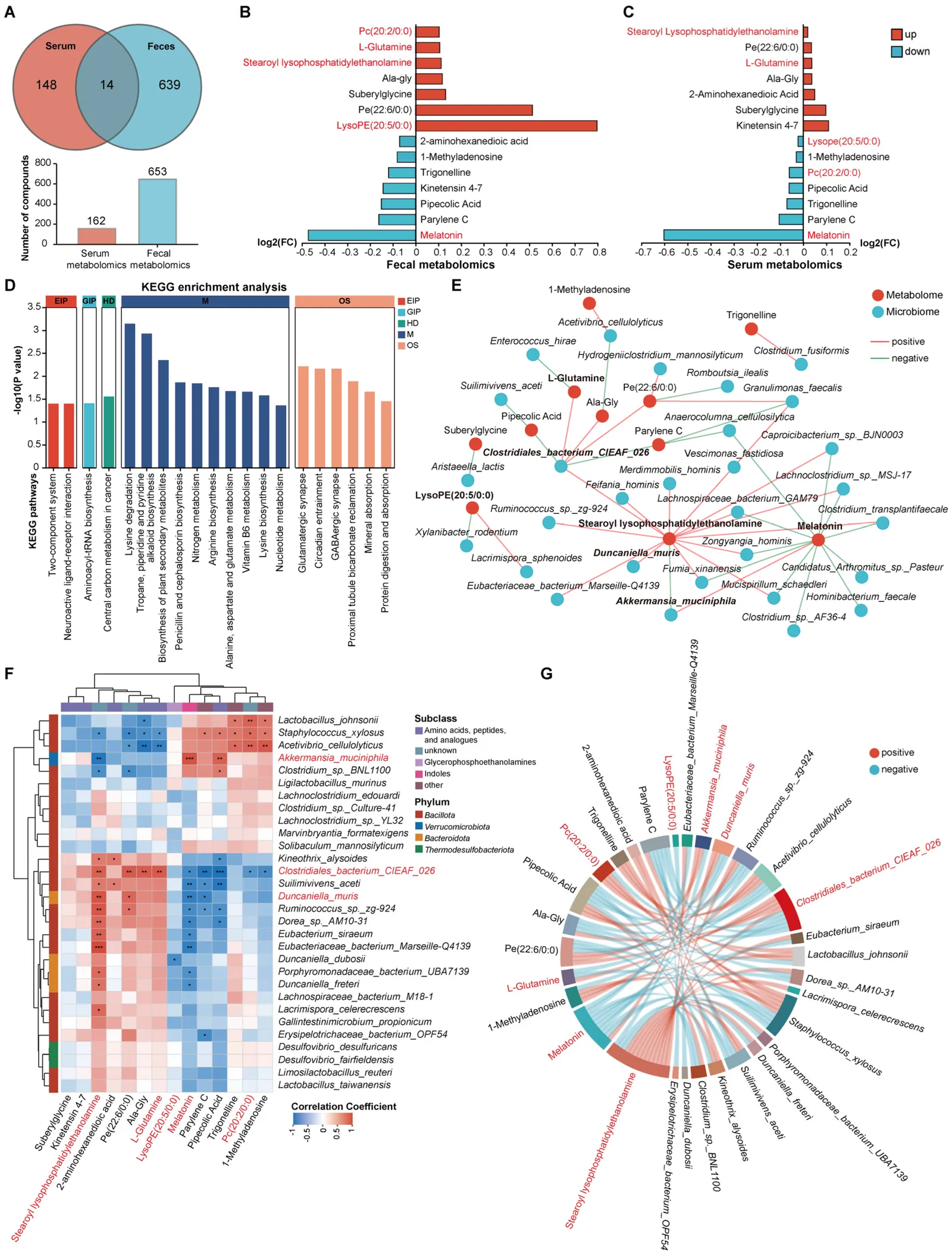
Integrated analysis of fecal and serum metabolomes reveals microbiota-metabolite interactions following CIH exposure. (**A**) Venn diagram showing overlapping differential metabolites identified from fecal and serum metabolomic datasets. (**B**,**C**) Fold-change analysis of the 14 shared differential metabolites in fecal (**B**) and serum (**C**) samples. (**D**) KEGG pathway enrichment analysis of the overlapping differential metabolites. (**E**) Correlation network depicting associations between differential gut microbial taxa and core metabolites (|r| > 0.8, *p* < 0.05). (**F**) Hierarchical clustering heatmap showing microbiota-metabolite correlation patterns. (**G**) Circos plot illustrating the relationships between representative microbial taxa and metabolites. *n* = 5 per group. * *p* < 0.05, ** *p* < 0.01, *** *p* < 0.001.

Following the integration of fecal and serum metabolomic datasets, 14 overlapping metabolites were identified between the two compartments (Figure 5A). Among them, stearoyl lysophosphatidylethanolamine (18:0 LysoPE) and L-glutamine were increased in both feces and serum, whereas melatonin was consistently decreased following CIH exposure. Notably, two phospholipid-related metabolites, PC (20:2/0:0) and LysoPE (20:5/0:0), exhibited opposite alterations between compartments, with increased levels in feces but reduced levels in serum, suggesting CIH-induced intestinal phospholipid remodeling and altered systemic lipid metabolism (Figure 5B,C).

KEGG enrichment analysis of these core metabolites revealed predominant involvement in amino acid and nitrogen metabolism, with lysine degradation showing the strongest enrichment significance (−log_10_(*p* value) > 3). In addition, neuroactive and circadian-related pathways, including glutamatergic synapse, GABAergic synapse, and circadian entrainment, were also enriched, suggesting potential links between CIH-associated metabolic disturbances and neurophysiological regulation (Figure 5D).

To investigate microbiota-metabolite interactions, Spearman correlation analysis was performed between the 14 core metabolites and differential gut microbial taxa (Figure 5E–G). *Clostridiales bacterium CIEAF 026* and *Duncaniella muris* were positively correlated with 18:0 LysoPE but negatively correlated with melatonin levels. In contrast, *A. muciniphila*, which was significantly depleted following CIH exposure, exhibited negative correlations with CIH-associated lipid metabolites, including 18:0 LysoPE. Notably, *A. muciniphila* showed strong positive correlations with melatonin and PC (20:2/0:0). Although several microbiota-metabolite associations met the predefined nominal significance criteria, some did not remain significant after Benjamini–Hochberg FDR correction, which may reflect the limited sample size and the reduced statistical power associated with multiple-testing correction. These findings suggest that CIH is associated with a disrupted gut microbiota-metabolite network, particularly involving alterations in glycerophospholipid metabolism and melatonin levels. However, these observed correlations should be interpreted as associative rather than causal.

### 3.5. CIH Disrupts Intestinal Barrier Integrity by Reducing Tight Junction Protein Expression

**Figure 6:**
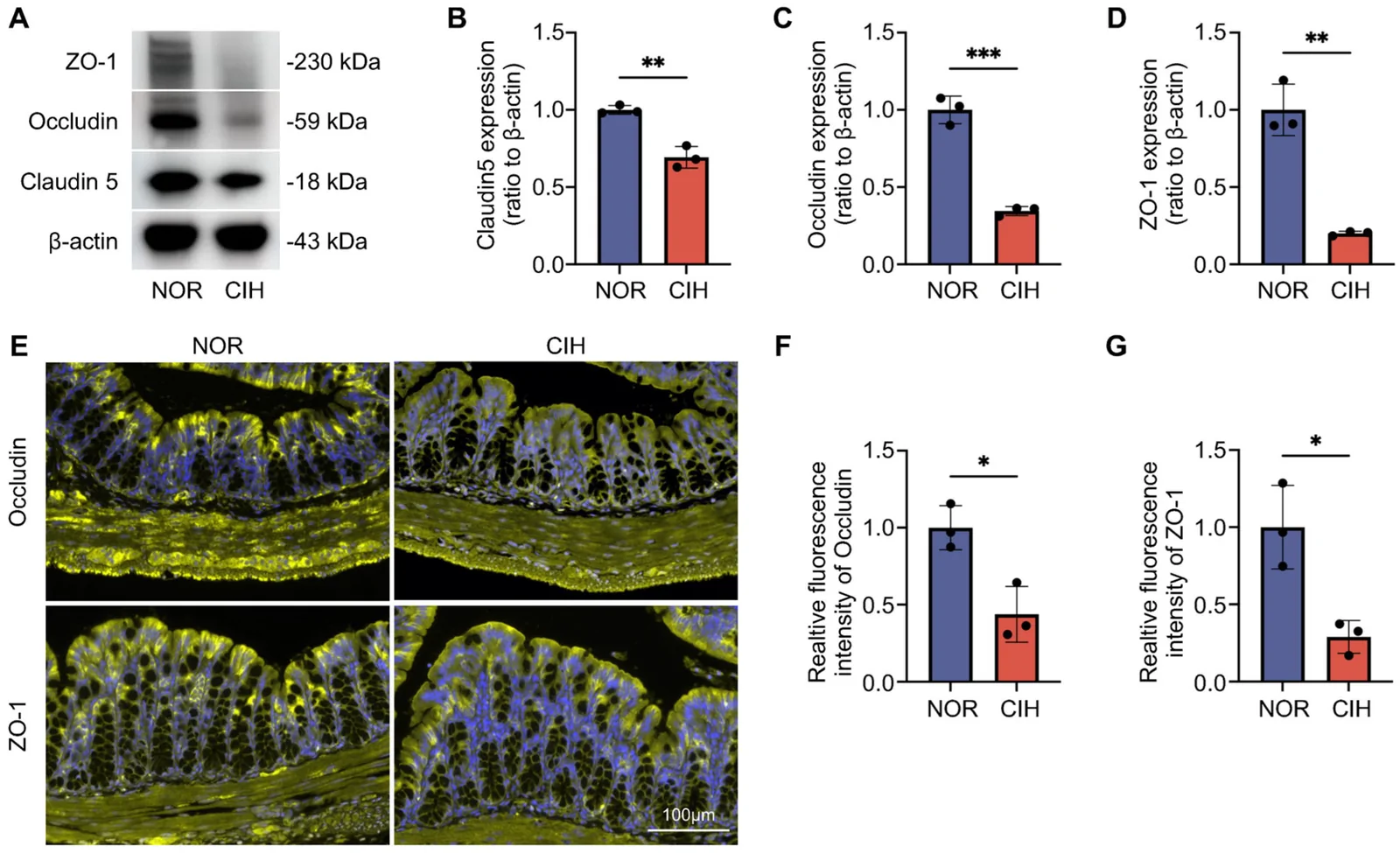
CIH impairs colonic tight junction integrity. (**A**) Representative Western blot images showing the expression of tight junction proteins in colonic tissues from NOR and CIH mice. (**B**–**D**) Quantification of ZO-1 (**B**), Occludin (**C**), and Claudin-5 (**D**) protein levels normalized to β-actin. (**E**) Representative immunofluorescence images showing ZO-1 and Occludin staining in colonic epithelial tissues. (**F**,**G**) Quantification of ZO-1 and Occludin fluorescence intensity. *n* = 3 per group. Data are presented as mean ± SD. * *p* < 0.05, ** *p* < 0.01, *** *p* < 0.001.

We next investigated whether CIH-induced remodeling of the gut microbiota and metabolic profile was accompanied by alterations in intestinal tight junction function. The expression of tight junction proteins was assessed by Western blotting and immunofluorescence. Western blot analysis demonstrated that the protein levels of ZO-1, Occludin, and Claudin-5 were significantly reduced in the colonic tissues of CIH mice compared with the NOR group (Figure 6A–D). Original Western Blot images for Figure 6A are found in Figure S1. Consistent with these findings, immunofluorescence staining revealed markedly weaker ZO-1 and Occludin signals in the colonic epithelium of CIH mice, indicating disrupted localization and decreased expression of these junctional proteins (Figure 6E). Quantitative fluorescence analysis further confirmed a significant reduction in the fluorescence intensity of both ZO-1 and Occludin following CIH exposure (Figure 6F). These results demonstrate that CIH compromises intestinal epithelial tight junctions and impairs intestinal barrier integrity.

## 4. Discussion

By integrating histological assessment, gut microbiome profiling, fecal and serum metabolomics, and multi-omics correlation analysis, the present study provides a comprehensive view of intestinal alterations induced by CIH. Our findings demonstrate that CIH disrupts intestinal homeostasis by impairing the mucus and epithelial barriers, reshaping the gut microbiota, and inducing coordinated local and systemic metabolic remodeling. These results support a potential microbiota–metabolite–barrier axis linking intermittent hypoxia exposure to intestinal dysfunction.

One of the principal findings of this study is that six weeks of CIH exposure compromises colonic barrier integrity. Previous studies in experimental models of OSA have demonstrated that intermittent hypoxia increases intestinal permeability, promotes bacterial translocation, and contributes to systemic inflammation [10,15]. Consistent with these observations, our findings indicate that CIH impaired both the mucus barrier and the epithelial mechanical barrier, as supported by histological alterations and reduced tight-junction protein expression. Several mechanisms may contribute to CIH-mediated barrier disruption. Repetitive hypoxia–reoxygenation cycles can induce excessive ROS production, particularly through NADPH oxidase activation, thereby promoting oxidative stress and inflammatory signaling through pathways such as NF-κB and MAPK [27,28]. Activation of these pathways can increase the production of pro-inflammatory mediators and promote tight junction disassembly, ultimately increasing epithelial permeability. In parallel, experimental intermittent hypoxia has also been shown to increase intestinal HIF-1α expression, accompanied by activation of NF-κB and reduced expression of tight junction proteins, including claudin-1 and claudin-4, together with morphological mucosal injury [29,30].

However, oxidative stress and hypoxic injury alone may not fully explain the intestinal abnormalities observed after CIH exposure. Increasing evidence suggests that the gut microbiota represents a critical regulator of epithelial integrity and mucosal homeostasis [31]. The intestinal microbial ecosystem is highly dependent on the oxygen gradient and nutrient availability within the gut environment, which maintains the dominance of anaerobic commensals and supports host–microbe interactions [8,32,33]. Consistent with previous reports in both experimental CIH models and patients with OSA [15,34,35], our microbiome analysis demonstrated that CIH markedly remodeled the intestinal microbial community.

Importantly, *A. muciniphila* was significantly depleted following CIH exposure. As a key mucus-associated commensal bacterium, *A. muciniphila* colonizes the inner mucus layer and utilizes mucin-derived substrates to maintain mucus turnover and epithelial protection [18]. Through production of beneficial metabolites, including short-chain fatty acids, *A. muciniphila* promotes goblet cell function, enhances tight junction integrity, and suppresses inflammatory responses [36]. Thus, the depletion of *A. muciniphila* following CIH exposure may be associated with impaired mucus homeostasis. Conversely, disruption of the mucus layer could alter the ecological niche and substrate availability for *A. muciniphila*, providing a possible explanation for the observed association between mucus loss and *A. muciniphila* depletion.

Because microbial metabolites may contribute to the functional effects of microbial dysbiosis [37], we integrated microbiome and metabolomic datasets to identify metabolites that may be associated with microbial alterations and host responses. Consistent with previous studies showing that intermittent hypoxia affects lipid metabolism, amino acid metabolism, and oxidative stress-related pathways [23,38,39], our results revealed extensive metabolic remodeling in both fecal and serum samples.

Among the altered metabolites, glycerophospholipid species were identified as important differential metabolites within the microbiota-metabolite network, which is consistent with previous studies demonstrating the pivotal role of lipid metabolism in gut microbiota–host interactions [23]. Glycerophospholipids are essential structural components of cellular membranes and function as bioactive mediators involved in inflammation, oxidative stress responses, and epithelial repair [40]. Notably, PC (20:2/0:0) and LysoPE (20:5/0:0) exhibited opposite patterns between fecal and serum compartments, with increased abundance in feces but decreased levels in circulation. This compartment-specific redistribution may reflect impaired intestinal absorption caused by epithelial barrier disruption or altered microbial lipid metabolism within the intestinal lumen. Although further mechanistic studies are required, these findings suggest that CIH induces spatially distinct metabolic remodeling rather than uniform systemic metabolic changes. The phospholipid abnormalities observed in the present study may also have implications beyond intestinal pathology. Dysregulated phosphatidylcholine and lysophospholipid metabolism have been implicated in numerous OSA-associated comorbidities, including obesity, insulin resistance, non-alcoholic fatty liver disease, and cardiovascular disease [23,41,42]. Clinical metabolomic studies have also identified disturbed glycerophospholipids metabolism in patients with OSA, including lysophospholipids, phosphatidic acid, phosphatidylcholine, phosphatidylserine, and cardiolipin [43]. In addition, altered circulating phosphatidylcholine species have been reported in patients with metabolic syndrome and atherosclerosis [44,45]. Although the specific lipid species were not completely concordant between our CIH model and human OSA cohorts, the overlap at the level of glycerophospholipid metabolism supports the potential translational relevance of our findings to the systemic metabolic alterations associated with OSA.

Beyond lipid metabolism, our study identified a consistent reduction in melatonin levels in both fecal and serum samples after CIH exposure. Melatonin is not only a central regulator of circadian rhythm but also an important modulator of intestinal barrier integrity, oxidative balance, and immune homeostasis [46,47,48]. The gastrointestinal tract contains substantial amounts of melatonin and has the capacity for local melatonin synthesis, with melatonin and its biosynthetic enzymes detected in enterochromaffin (EC) cells [49]. In the gastrointestinal tract, melatonin contributes to epithelial renewal, mucus secretion, and tight-junction maintenance, supporting intestinal barrier homeostasis [47]. Recurrent hypoxia, sleep fragmentation, and sympathetic activation have all been proposed to contribute to altered melatonin synthesis and secretion [50]. Our integrated microbiome–metabolome analysis further demonstrated that melatonin levels were positively correlated with *A. muciniphila* abundance but negatively associated with several CIH-enriched bacterial taxa, consistent with previous evidence suggesting an intricate functional relationship between melatonin and the intestinal microbiota [51]. The reduction in melatonin observed after CIH exposure may therefore reflect a direct effect of intermittent hypoxia on melatonin production or an indirect effect of microbiota alterations, or a combination of both mechanisms. Experimental studies further demonstrated that exogenous melatonin supplementation attenuates oxidative stress, restores tight junction protein expression, alleviates intestinal inflammation, and partially reverses intestinal barrier dysfunction induced by CIH [10,52]. Taken together, these findings suggest a potential interaction between gut microbiota and intestinal melatonin homeostasis in the context of CIH exposure.

Furthermore, KEGG enrichment analysis based on metabolites shared between the fecal and serum metabolomic profiles revealed alterations in pathways related to circadian entrainment and neuroactive signaling, including glutamatergic and GABAergic synapse pathways. Glutamate and GABA are major excitatory and inhibitory neurotransmitters, respectively, and their metabolism has been implicated in gut–brain communication [53]. The enrichment of these pathways among metabolites detected in both fecal and serum samples suggests that CIH-related metabolic alterations may be relevant to neuroactive signaling between the gut and systemic circulation. Although these pathway-level predictions require further experimental validation, these findings raise the possibility that intestinal metabolic disturbances associated with CIH may have broader implications for gut–brain communication and the neurophysiological consequences of OSA.

The key strength of our study is that it provides a conceptual framework linking CIH exposure with gut microbiota remodeling, metabolic alterations, and intestinal barrier dysfunction. Nevertheless, several limitations should be acknowledged. First, the functional intestinal barrier impairment was not directly assessed. Future studies incorporating inflammatory cytokine profiling and functional permeability assays will be needed to further validate CIH-induced intestinal barrier dysfunction. Second, the causal relationships underlying this axis remain to be established. Future studies employing fecal microbiota transplantation, *A. muciniphila* supplementation, or targeted metabolite interventions will be necessary to validate the functional contributions of these factors. Third, the metabolomic findings were generated using untargeted LC–MS profiling, and the key metabolites identified here require further targeted quantitative validation. In addition, the molecular mechanisms through which microbiota-derived metabolites regulate intestinal barrier function and neuroendocrine signaling remain to be elucidated.

Despite these limitations, our study provides a comprehensive multi-omics characterization of intestinal alterations induced by CIH. By integrating histological evaluation with gut microbiome profiling and fecal–serum metabolomics, we identified depletion of *A. muciniphila*, phospholipid remodeling, and reduced melatonin levels as prominent features associated with CIH-induced intestinal dysfunction.

## 5. Conclusions

In conclusion, the present study demonstrates that CIH induces intestinal dysfunction characterized by impairment of the mucus and epithelial barriers, gut microbial dysbiosis, and coordinated metabolic remodeling in both the intestinal lumen and systemic circulation. Multi-omics integration further identified depletion of *A. muciniphila*, altered glycerophospholipid metabolism, and reduced melatonin levels as key features associated with CIH-induced intestinal injury. These findings support a proposed microbiota–metabolite–barrier framework linking CIH exposure to intestinal dysfunction, although the causal relationships among these components remain to be experimentally validated. Given the observational nature of the present study, future studies using targeted metabolomic validation, fecal microbiota transplantation, and interventions targeting *A. muciniphila*, melatonin, or specific microbial metabolites are needed to determine their functional contributions. Clinical studies are also needed to determine whether similar intestinal and metabolic alterations occur in patients with OSA and to assess their clinical relevance. Collectively, these findings highlight the gut microbiota and specific microbial metabolites as potentially modifiable targets and suggest that restoring intestinal homeostasis may represent a promising adjunctive therapeutic strategy for mitigating the systemic consequences of OSA.

## Data Availability

Data are contained within the article and Supplementary Materials. The 16S rRNA sequencing data generated in this study have been deposited in the NCBI Sequence Read Archive (SRA) under BioProject accession number PRJNA1497099.

## Supplementary Materials

The following supporting information can be downloaded at https://www.mdpi.com/article/10.3390/biom16081186/s1, Figure S1: Original Western Blots images for Figure 6A.

## References

[1] Lv R., Liu X., Zhang Y., Dong N., Wang X., He Y., Yue H., Yin Q. (2023). Pathophysiological Mechanisms and Therapeutic Approaches in Obstructive Sleep Apnea Syndrome. Signal Transduct. Target. Ther..

[2] Lavie L. (2015). Oxidative Stress in Obstructive Sleep Apnea and Intermittent Hypoxia—Revisited—The Bad Ugly and Good: Implications to the Heart and Brain. Sleep Med. Rev..

[3] Senaratna C.V., Perret J.L., Lodge C.J., Lowe A.J., Campbell B.E., Matheson M.C., Hamilton G.S., Dharmage S.C. (2017). Prevalence of Obstructive Sleep Apnea in the General Population: A Systematic Review. Sleep Med. Rev..

[4] Ayyaswamy S., Shi H., Zhang B., Bryan R.M., Durgan D.J. (2023). Obstructive Sleep Apnea-Induced Hypertension Is Associated With Increased Gut and Neuroinflammation. J. Am. Heart Assoc..

[5] Badran M., Khalyfa A., Ericsson A.C., Puech C., McAdams Z., Bender S.B., Gozal D. (2023). Gut Microbiota Mediate Vascular Dysfunction in a Murine Model of Sleep Apnoea: Effect of Probiotics. Eur. Respir. J..

[6] Nicholson J.K., Holmes E., Kinross J., Burcelin R., Gibson G., Jia W., Pettersson S. (2012). Host-Gut Microbiota Metabolic Interactions. Science.

[7] Singhal R., Shah Y.M. (2020). Oxygen Battle in the Gut: Hypoxia and Hypoxia-Inducible Factors in Metabolic and Inflammatory Responses in the Intestine. J. Biol. Chem..

[8] Zheng L., Kelly C.J., Colgan S.P. (2015). Physiologic Hypoxia and Oxygen Homeostasis in the Healthy Intestine. A Review in the Theme: Cellular Responses to Hypoxia. Am. J. Physiol. Cell Physiol..

[9] Lee P., Chandel N.S., Simon M.C. (2020). Cellular Adaptation to Hypoxia through Hypoxia Inducible Factors and Beyond. Nat. Rev. Mol. Cell Biol..

[10] Li X., Wang F., Gao Z., Huang W., Zhang X., Liu F., Yi H., Guan J., Wu X., Xu H. (2023). Melatonin Attenuates Chronic Intermittent Hypoxia-Induced Intestinal Barrier Dysfunction in Mice. Microbiol. Res..

[11] Wang P.-P., Cheng X.-Q., Dou Z.-J., Fan Y.-Q., Chen J., Zhao L., Han J.-X., Lin X.-W., Wang B. (2024). Inhibiting the CB1 Receptor in CIH-Induced Animal Model Alleviates Colon Injury. Appl. Microbiol. Biotechnol..

[12] Mashaqi S., Rangan P., Saleh A.A., Abraham I., Gozal D., Quan S.F., Parthasarathy S. (2023). Biomarkers of Gut Barrier Dysfunction in Obstructive Sleep Apnea: A Systematic Review and Meta-Analysis. Sleep Med. Rev..

[13] Ko C.-Y., Zhang L., Lin Y., Zeng Y. (2026). Gut Microbial Metabolites, Barrier Dysfunction, and Endotoxemia in Men Obstructive Sleep Apnea-Associated Hypertension. iScience.

[14] Pral L.P., Fachi J.L., Corrêa R.O., Colonna M., Vinolo M.A.R. (2021). Hypoxia and HIF-1 as Key Regulators of Gut Microbiota and Host Interactions. Trends Immunol..

[15] Moreno-Indias I., Torres M., Montserrat J.M., Sanchez-Alcoholado L., Cardona F., Tinahones F.J., Gozal D., Poroyko V.A., Navajas D., Queipo-Ortuño M.I. (2015). Intermittent Hypoxia Alters Gut Microbiota Diversity in a Mouse Model of Sleep Apnoea. Eur. Respir. J..

[16] O’Connor K.M., Lucking E.F., Bastiaanssen T.F.S., Peterson V.L., Crispie F., Cotter P.D., Clarke G., Cryan J.F., O’Halloran K.D. (2020). Prebiotic Administration Modulates Gut Microbiota and Faecal Short-Chain Fatty Acid Concentrations but Does Not Prevent Chronic Intermittent Hypoxia-Induced Apnoea and Hypertension in Adult Rats. EBioMedicine.

[17] Sun T., Sun R., Yan J., Luo L., Que M., Wang X., Zhang Z., Li S., Zhou Z., Zhao Y. (2026). Bifidobacterium Pseudolongum Alleviates Chronic Intermittent Hypoxia-Induced Cognitive Impairment by Restoring Acetate Metabolism and Suppressing Hippocampal Neuroinflammation and Neuronal PANoptosis. Microbiol. Res..

[18] Ioannou A., Berkhout M.D., Geerlings S.Y., Belzer C. (2025). Akkermansia Muciniphila: Biology, Microbial Ecology, Host Interactions and Therapeutic Potential. Nat. Rev. Microbiol..

[19] Panzetta M.E., Valdivia R.H. (2024). Akkermansia in the Gastrointestinal Tract as a Modifier of Human Health. Gut Microbes.

[20] Kelly C.J., Zheng L., Campbell E.L., Saeedi B., Scholz C.C., Bayless A.J., Wilson K.E., Glover L.E., Kominsky D.J., Magnuson A. (2015). Crosstalk between Microbiota-Derived Short-Chain Fatty Acids and Intestinal Epithelial HIF Augments Tissue Barrier Function. Cell Host Microbe.

[21] Gasaly N., de Vos P., Hermoso M.A. (2021). Impact of Bacterial Metabolites on Gut Barrier Function and Host Immunity: A Focus on Bacterial Metabolism and Its Relevance for Intestinal Inflammation. Front. Immunol..

[22] Mann E.R., Lam Y.K., Uhlig H.H. (2024). Short-Chain Fatty Acids: Linking Diet, the Microbiome and Immunity. Nat. Rev. Immunol..

[23] Zhang X., Wang S., Xu H., Yi H., Guan J., Yin S. (2021). Metabolomics and Microbiome Profiling as Biomarkers in Obstructive Sleep Apnoea: A Comprehensive Review. Eur. Respir. Rev..

[24] Di Vincenzo F., Del Gaudio A., Petito V., Lopetuso L.R., Scaldaferri F. (2024). Gut Microbiota, Intestinal Permeability, and Systemic Inflammation: A Narrative Review. Intern. Emerg. Med..

[25] Wu Z., Zhai M., Wang Y., Ren L., Pan J., Xiao L., Liu Y. (2025). Astrocyte-Microglia Crosstalk in the Hippocampus Mediates Cognitive Impairments Induced by Chronic Intermittent Hypoxia. Neurobiol. Dis..

[26] Zhai M.-R., Pan J., Wu Z.-H., He Y.-Y., Zhang K.-R., Ren L., Wang Y.-R., Li Y.-B., Gao J., Xiao L. (2026). Chronic Intermittent Hypoxia Increases Parkinson’s Disease Susceptibility via PPARα-Mediated Lipid Droplet-Mitochondrial Dysfunction. Theranostics.

[27] Badran M., Cortese R., Gileles-Hillel A., Gozal D. (2026). Mechanisms Underlying End-Organ Injury in Sleep Apnoea. Eur. Respir. J..

[28] Makarenko V.V., Usatyuk P.V., Yuan G., Lee M.M., Nanduri J., Natarajan V., Kumar G.K., Prabhakar N.R. (2014). Intermittent Hypoxia-Induced Endothelial Barrier Dysfunction Requires ROS-Dependent MAP Kinase Activation. Am. J. Physiol. Cell Physiol..

[29] Wu J., Sun X., Wu Q., Li H., Li L., Feng J., Zhang S., Xu L., Li K., Li X. (2016). Disrupted Intestinal Structure in a Rat Model of Intermittent Hypoxia. Mol. Med. Rep..

[30] Steiner C.A., Cartwright I.M., Taylor C.T., Colgan S.P. (2022). Hypoxia-Inducible Factor as a Bridge between Healthy Barrier Function, Wound Healing, and Fibrosis. Am. J. Physiol. Cell Physiol..

[31] Turner J.R. (2009). Intestinal Mucosal Barrier Function in Health and Disease. Nat. Rev. Immunol..

[32] Wang T., Wang R.X., Colgan S.P. (2024). Physiologic Hypoxia in the Intestinal Mucosa: A Central Role for Short-Chain Fatty Acids. Am. J. Physiol. Cell Physiol..

[33] Glover L.E., Lee J.S., Colgan S.P. (2016). Oxygen Metabolism and Barrier Regulation in the Intestinal Mucosa. J. Clin. Investig..

[34] Moreno-Indias I., Torres M., Sanchez-Alcoholado L., Cardona F., Almendros I., Gozal D., Montserrat J.M., Queipo-Ortuño M.I., Farré R. (2016). Normoxic Recovery Mimicking Treatment of Sleep Apnea Does Not Reverse Intermittent Hypoxia-Induced Bacterial Dysbiosis and Low-Grade Endotoxemia in Mice. Sleep.

[35] Ko C.-Y., Liu Q.-Q., Su H.-Z., Zhang H.-P., Fan J.-M., Yang J.-H., Hu A.-K., Liu Y.-Q., Chou D., Zeng Y.-M. (2019). Gut Microbiota in Obstructive Sleep Apnea-Hypopnea Syndrome: Disease-Related Dysbiosis and Metabolic Comorbidities. Clin. Sci..

[36] Kim S., Shin Y.-C., Kim T.-Y., Kim Y., Lee Y.-S., Lee S.-H., Kim M.-N., O E., Kim K.S., Kweon M.-N. (2021). Mucin Degrader Akkermansia Muciniphila Accelerates Intestinal Stem Cell-Mediated Epithelial Development. Gut Microbes.

[37] Krautkramer K.A., Fan J., Bäckhed F. (2021). Gut Microbial Metabolites as Multi-Kingdom Intermediates. Nat. Rev. Microbiol..

[38] Tripathi A., Melnik A.V., Xue J., Poulsen O., Meehan M.J., Humphrey G., Jiang L., Ackermann G., McDonald D., Zhou D. (2018). Intermittent Hypoxia and Hypercapnia, a Hallmark of Obstructive Sleep Apnea, Alters the Gut Microbiome and Metabolome. mSystems.

[39] Barros D., García-Río F. (2019). Obstructive Sleep Apnea and Dyslipidemia: From Animal Models to Clinical Evidence. Sleep.

[40] Mauerhofer C., Philippova M., Oskolkova O.V., Bochkov V.N. (2016). Hormetic and Anti-Inflammatory Properties of Oxidized Phospholipids. Mol. Asp. Med..

[41] Drager L.F., Togeiro S.M., Polotsky V.Y., Lorenzi-Filho G. (2013). Obstructive Sleep Apnea: A Cardiometabolic Risk in Obesity and the Metabolic Syndrome. J. Am. Coll. Cardiol..

[42] Rhee E.P., Cheng S., Larson M.G., Walford G.A., Lewis G.D., McCabe E., Yang E., Farrell L., Fox C.S., O’Donnell C.J. (2011). Lipid Profiling Identifies a Triacylglycerol Signature of Insulin Resistance and Improves Diabetes Prediction in Humans. J. Clin. Investig..

[43] Pinilla L., Benítez I.D., Gracia-Lavedan E., Torres G., Mínguez O., Vaca R., Jové M., Sol J., Pamplona R., Barbé F. (2023). Metabolipidomic Analysis in Patients with Obstructive Sleep Apnea Discloses a Circulating Metabotype of Non-Dipping Blood Pressure. Antioxidants.

[44] Stegemann C., Pechlaner R., Willeit P., Langley S.R., Mangino M., Mayr U., Menni C., Moayyeri A., Santer P., Rungger G. (2014). Lipidomics Profiling and Risk of Cardiovascular Disease in the Prospective Population-Based Bruneck Study. Circulation.

[45] Alshehry Z.H., Mundra P.A., Barlow C.K., Mellett N.A., Wong G., McConville M.J., Simes J., Tonkin A.M., Sullivan D.R., Barnes E.H. (2016). Plasma Lipidomic Profiles Improve on Traditional Risk Factors for the Prediction of Cardiovascular Events in Type 2 Diabetes Mellitus. Circulation.

[46] Cipolla-Neto J., Amaral F.G.D. (2018). Melatonin as a Hormone: New Physiological and Clinical Insights. Endocr. Rev..

[47] LeFort K.R., Rungratanawanich W., Song B.-J. (2023). Melatonin Prevents Alcohol- and Metabolic Dysfunction- Associated Steatotic Liver Disease by Mitigating Gut Dysbiosis, Intestinal Barrier Dysfunction, and Endotoxemia. Antioxidants.

[48] Wei Z., Shen H., Wang F., Huang W., Li X., Xu H., Zhu H., Guan J. (2024). Melatonin Mediates Intestinal Barrier Dysfunction and Systemic Inflammation in Moderate-Severe OSA Patients. Ann. Med..

[49] Bubenik G.A. (2001). Localization, Physiological Significance and Possible Clinical Implication of Gastrointestinal Melatonin. Neurosignals.

[50] Hernández C., Abreu J., Abreu P., Castro A., Jiménez A. (2007). Nocturnal Melatonin Plasma Levels in Patients with OSAS: The Effect of CPAP. Eur. Respir. J..

[51] Iesanu M.I., Zahiu C.D.M., Dogaru I.-A., Chitimus D.M., Pircalabioru G.G., Voiculescu S.E., Isac S., Galos F., Pavel B., O’Mahony S.M. (2022). Melatonin-Microbiome Two-Sided Interaction in Dysbiosis-Associated Conditions. Antioxidants.

[52] Gao T., Wang Z., Dong Y., Cao J., Lin R., Wang X., Yu Z., Chen Y. (2019). Role of Melatonin in Sleep Deprivation-Induced Intestinal Barrier Dysfunction in Mice. J. Pineal Res..

[53] Baj A., Moro E., Bistoletti M., Orlandi V., Crema F., Giaroni C. (2019). Glutamatergic Signaling Along The Microbiota-Gut-Brain Axis. Int. J. Mol. Sci..

