# Supplementary material for: Chronic Intermittent Hypoxia Disrupts Intestinal Homeostasis Through Gut Microbiota Remodeling and Microbiota-Metabolite Interactions
Source: Biomolecules. 2026 Aug 14;16(8):1186. doi: 10.3390/biom16081186 (PMC13510920; doi:10.3390/biom16081186)
Supplement: Supplementary file 1 [file biomolecules-16-01186-s001.zip › biomolecules-4476510-supplementary.pdf]

biomolecules

MDPI

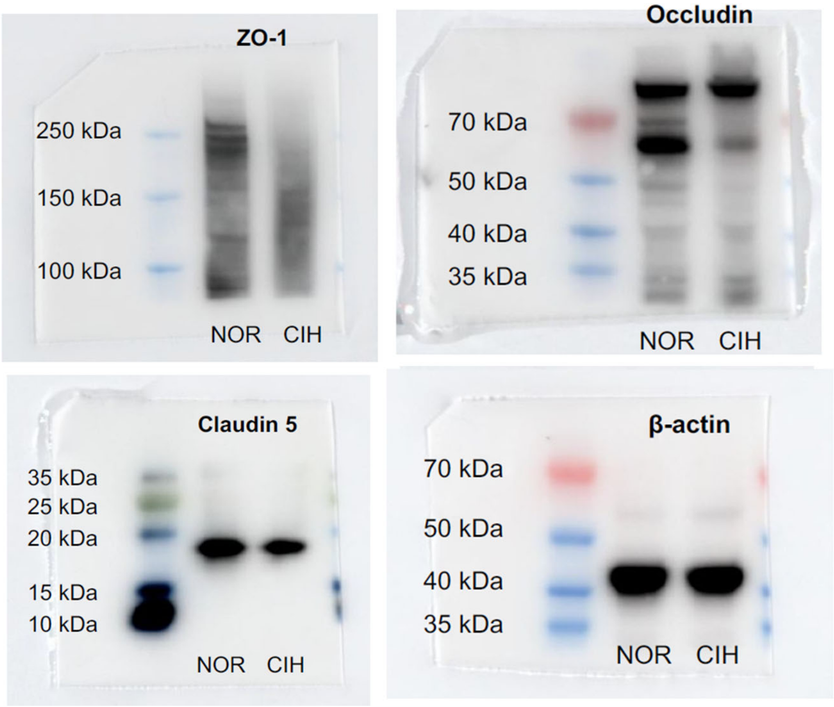

Figure S1. Original Western Blots images for Figure 6A.
